# P-1350. Novel mutations in PBP1 and AcrB associated with methicillin resistance in clinical isolates of *S. aureus* lacking *mec* variants

**DOI:** 10.1093/ofid/ofae631.1527

**Published:** 2025-01-29

**Authors:** Maria Alejandra Mancera, Juan Matiz, Aura Lucia Leal Castro, Rodrigo de Paula Baptista, Sandra Rincon, Lina Paola Carvajal, Adriana Marcela Celis Ramirez, Jinnethe Reyes

**Affiliations:** Universidad de los Andes, Bogotá, Distrito Capital de Bogota, Colombia; Universidad el Bosque, Bogotá, Distrito Capital de Bogota, Colombia; Universidad Nacional de Colombia, Bogotá, Distrito Capital de Bogota, Colombia; Houston Methodist Hospital, Houston, Texas; Molecular Genetics and Antimicrobial Resistance Unit and International Center of Microbial Genomics, Universidad El Bosque, Bogota, Colombia, Bogota, Distrito Capital de Bogota, Colombia; Universidad El Bosque, Bogotá D.C., Distrito Capital de Bogota, Colombia; Universidad de los Andes, Bogotá, Distrito Capital de Bogota, Colombia; Molecular Genetics and Antimicrobial Resistance Unit, Universidad El Bosque, Bogota, Distrito Capital de Bogota, Colombia

## Abstract

**Background:**

Methicillin-resistant *S. aureus* (MRSA) is a major global pathogen. In the absence of *mecA* and *mecC*, methicillin resistance has been associated with genetic changes, such as PBP1 to PBP4 (Penicillin-Binding Protein) and GdpP (c-di-AMP phosphodiesterase) mutations. However, this unusual genotype has not been reported in Colombia. Here, the aim was to characterize the genetic determinants in two methicillin-resistant clinical isolates of *S. aureus* lacking *mec* variants in Colombia.

Table 1

**Figure:**
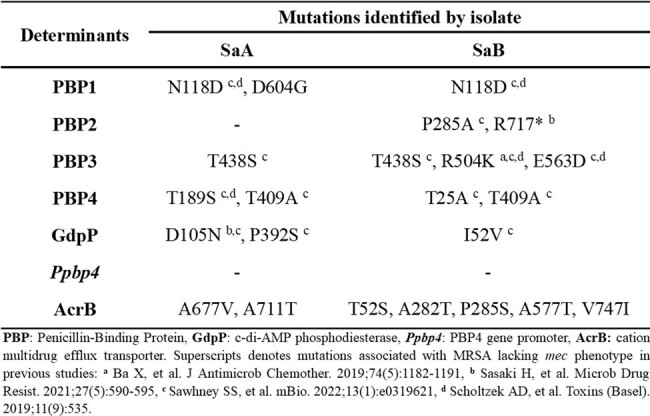

Genetic changes associated with methicillin resistance lacking mec variants in SaA and SaB isolates.

**Methods:**

One isolate (SaA) was recovered in 2015 from a urine culture and the second (SaB) was obtained in 2016 from a peritoneal fluid secretion. OXA susceptibility was assessed via broth microdilution according to CLSI M100 guidelines, and species confirmation and *mecA* detection were performed using multiplex PCR. Genome characterization by Illumina Whole Genome Sequencing was conducted to evaluate the presence of *mec* variants (A, B, or C) and determinants associated with OXA resistance lacking *mec* variants (mutations in PBP1 to PBP4, *pbp4* promoter, GdpP, and AcrB).

**Results:**

Both isolates were identified as MRSA (OXA MIC of 16 ug/mL) but lacked *mecA* by PCR. In addition, genomic characterization showed that SaA (ST-5/CC-5/*agr*-III) and SaB (ST-765/CC-30/*agr*-I) do not harbor any *mec* variants. Moreover, the *bla* operon was complete in SaB, whereas was absent in SaA. Notably, we found genetic changes in SaA and SaB previously associated with methicillin resistance lacking *mec* (Table 1), some of which were common in both isolates (PBP1: N118D, PBP3: T438S and PBP4: T409A). Mutations in PBP2 were only detected in SaB. Remarkably, novel mutations, such as D604G in PBP1 and AcrB mutations (T52S, A282T, P285S, A577T, A677V, A711T, and V747I) were identified (Table 1).

**Conclusion:**

Novel mutations in PBP1 and AcrB were found in clinical isolates of MRSA lacking *mec* variants, as well as previously documented genetic changes. This emergent unusual genotype needs further investigation.

**Disclosures:**

**All Authors**: No reported disclosures

